# Phytochemical Composition, Antioxidant, and Enzyme Inhibition Activities of Methanolic Extracts of Two Endemic *Onosma* Species

**DOI:** 10.3390/plants10071373

**Published:** 2021-07-05

**Authors:** Kandasamy Saravanakumar, Cengiz Sarikurkcu, Saliha Seyma Sahinler, Rabia Busra Sarikurkcu, Myeong-Hyeon Wang

**Affiliations:** 1 Department of Bio Health Convergence, Kangwon National University, Chuncheon 24341, Gangwon, Korea; saravana732@gmail.com or; 2 Department of Analytical Chemistry, Faculty of Pharmacy, Afyonkarahisar Health Sciences University, Afyonkarahisar 03100, Turkey; 3 Department of Pharmacognosy, Faculty of Pharmacy, Afyonkarahisar Health Sciences University, Afyonkarahisar 03100, Turkey; bsssahinler03@gmail.com; 4 Department of Coaching Education, Faculty of Sport Sciences, Ankara University, Golbasi Campus, Ankara 06830, Turkey; rbsarikurkcu03@gmail.com

**Keywords:** *Onosma* species, phyto-compounds, antioxidants, enzyme inhibitors

## Abstract

*Onosma* species have been used as a dye for hundreds of years due to their dark red pigments. These species have also been used by mankind in the treatment of various diseases since ancient times. This work analyzed the phytochemical composition in methanol extract of two endemic *Onosma* species (*O. lycaonica* and *O. papillosa*). Methanolic extract of these species varied in the content of flavonoids and phenolics. The flavonoids were found higher in *O. papillosa* [32.9 ± 0.3 mg QEs (quercetin equivalent)/g extracts] while the phenolics were higher in *O. lycaonica* [43.5 ± 1.5 mg GAEs (gallic acid equivalent)/g extracts]. ESI-MS/MS (electrospray ionization-mass spectrometry) revealed the presence of 25 compounds in *O. lycaonica* and 24 compounds in *O. papillosa*. The former was richer than the latter for apigenin, luteolin, eriodictyol, pinoresinol, apigenin 7-glucoside, rosmarinic acid, luteolin 7-glucoside, ferulic acid, vanillin, caffeic acid, 4-hydroxybenzoic acid, (+)-catechin3,4-dihydroxyphenylacetic acid. The *O. papillosa* exhibited low EC_50_ (1.90 ± 0.07 mg/mL) which indicated its strong phosphomolybdenum scavenging activity as compared to *O. lycaonica*. However, the *O. lycaonica* showed low IC_50_ or EC_50_ for 1,1-diphenyl-2-picrylhydrazyl (DPPH), 2,2′-azino-bis(3-ethylbenzothiazoline-6-sulfonic acid) (ABTS+), cupric reducing antioxidant power (CUPRAC), ferric reducing antioxidant power (FRAP) and ferrous ion chelating activity, as compared to *O. papillosa*. The results proved the presence of potent antioxidant compounds in *O. lycaonica*. Further, the plant extracts significantly varied for enzyme inhibition of acetylcholinesterase (AChE) and butyrylcholinesterase (BChE), but the plant extracts did not significantly differ for inhibition of α-glucosidase, α-amylase, and tyrosinase. *Onosma* species deserve further research towards developing novel drugs to treat oxidative diseases.

## 1. Introduction

*Onosma* genus contains species rich in shikonins. Particularly the red dye of *Onosma* sp., has been used since ancient times both in fabric dyeing (especially in silk dyeing) and a dyestuff in foods [1]. Also, this pigment offers alternative solutions for cosmetics and medicine due to its bioactivity [2,3]. Hence, researchers have been prompted to analyse the bioactivities of *Onosma* sp. [4]. Oxidative stress stimulates various diseases. Free radicals and reactive oxygen species are oxidants formed as intermediates during various metabolic functions. Oxidants are simultaneously scavenged by elevated synthesis of suitable enzymes (superoxidase dismutase, hydroperoxides, catalase). Therefore, a balance between oxidants and antioxidants is essential to ensure a healthy metabolic activity defense system. However, if unbalancing of oxidants and antioxidants occurs, will lead to the oxidative stress [5,6], which is known to cause various diseases including atherosclerosis, coronary heart disease and aging diseases (cancer, arthritis, osteoporosis, cataracts, type 2 diabetes, Alzheimer’s disease and hypertension) [7]. Several synthetic antioxidant molecules are consumed to prevent oxidative stress-related diseases but they cause adverse effects [8]. The consumption of antioxidant-rich foods such as green tea, fresh vegetables and fruits successfully prevents oxidative stress. Hence, it is essential to discover novel antioxidants from natural resources to erase the risk of oxidative stress-mediated diseases.

Earlier research findings have revealed that medicinal plants are promising among natural resources for the presence of antioxidants such as polyphenols, tannins, flavonoids, polysaccharides, ascorbic acid, tocopherols and anthocyanins [9]. Therefore, it is essential to understand the composition of these metabolites and their antioxidant properties [10]. Earlier studies have screened medicinal plants using primary antioxidant assays (phosphomolybdenum, DPPH, ABTS, CUPRAC, FRAP, ferrous ion chelating) and enzyme inhibition assays (AChE inhibition, BChE inhibition, α-amylase inhibition, α-glucosidase inhibition, and tyrosinase inhibition) [11,12,13]. These screening assays are used to relate the oxidative stress diseases, Alzheimer’s disease, photocarcinogenesis and diabetes mellitus, [14,15,16]. 

*Onosma* species (family Boraginaceae) are globally distributed medicinal plants with a total of 180 species. In Turkey, alone there are 85 species of *Onosma*, including more than 40 endemic species [17,18], and they are traditionally used in the country as natural remedies to cure fever, bladder pain, kidney infections, blood diseases, burns, abdominal pain and wound healing [19]. The phytochemistry and ethnopharmacology of several *Onosma* spp., have been documented, but not for two endemic *Onosma* spp., (*O. lycaonica* and *O. papillosa*). Hence, the present work analyzed the phenolic and flavonoid compounds from these *Onosma* spp. by using LC-ESI-MS/MS, and also tested their bioactivities in terms of antioxidation and enzyme inhibition properties.

*Onosma* species are important medicinal plants due to their promising pharmacological properties, including antioxidant, anti-inflammatory, cytotoxicity, and enzyme inhibition activities [20], Moreover, these plants are traditionally recognized as folk medicines to cure several aging-related diseases [19]. Generally antioxidant, antimicrobial, enzyme inhibitory, anti-inflammatory, and anti-cardiovascular disease biological activities are related to the phenolics, tannins and flavonoids present in plants [21]. It is essential to study the phytochemistry of plants to establish a basis for the isolation of novel compounds as drug candidates to treat the aforesaid diseases. Therefore, the present study examined the phytochemical composition in the methanol extract of two endemic *Onosma* species (*O. lycaonica* and *O. papillosa*) by spectrophotometric and LC-ESI-MS/MS methods.

## 2. Results and Discussion

### 2.1. Yield, Total Phenolics and Flavonoids

The two plant species significantly varied in total flavonoids and total phenolics (*p* < 0.05; Table 1). The yield was higher for the methanol extract of *O. papillosa* (4.02%) than *O. lycaonica* (3.52%). Similarly, the content of flavonoids was higher in *O. papillosa* (32.9 ± 0.3 mg QEs/g extract) than in *O. lycaonica* (26.0 ± 0.5 mg QEs/g extract). However, the content of phenolics was found higher in *O. lycaonica* (43.5 ± 1.5 mg GAEs/g extract) than in *O. papillosa* (33.9 ± 0.4 mg GAEs/g extract). These results revealed that *O. lycaonica* was rich in phenolics while *O. papillosa* was rich in flavonoids (Table 1). Several earlier studies have also reported varied levels of total phenolic and flavonoids in *Onosma* species (*O. stenoloba*, *O. sericea*, *O. isaurica*, *O. bracteosa*, *O. tauricum*, and *O. gigantea*) [11,12,22,23]. For example, the content of total phenolic is reportedly higher in *O.sericea* (69.8 ± 1.0 mg GAEs/g extract) than *O. stenoloba* (32.5 ± 0.6 mg GAEs/g extract) [12]. 

### 2.2. Phytochemical Composition 

**Figure 1:**
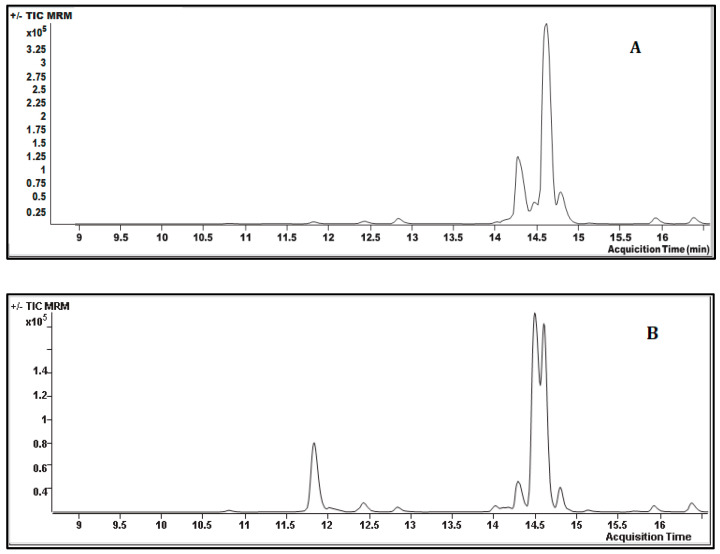
Liquid chromatography electrospray ionization tandem mass spectrometry (LC−ESI−MS/MS) chromatograms of the methanol extracts of (**A**) *Onosma lycaonica* and (**B**) *O. papillosa*.

Although ESI-MS/MS is a frequently used method for the quantification the phyto compounds, it is essential to standardize the operating conditions for sensitive target compounds based on their MRM ionization modes. Therefore, the present study standardized the analytical parameters of LC-ESI-MS/MS in response to negative and positive ionization using the standard molecules (Table S1). After the establishment of LC-MS operating conditions, a total of 31 standard flavonoids/phenolic compounds were used at different concentrations to prepare a standard curve. The results of standard compounds were fitted to the calibration curve, and their linear equations and R^2^ values are presented in the Supplementary Information (Table S2). The LC-MS/MS mediated quantification of phytocompounds in two *Onosma* species are presented in Table 2, which shows that out of 31 compounds studied, a total of 25 compounds were present in *O. lycaonica* while 24 compounds were observed in *O. papillosa* (Figure 1). Five compounds, including pyrocatechol, (−)-epicatechin, verbascoside, taxifolin and 2-hydroxycinnamic acid were absent in both *O. lycaonica* and *O. papillosa*. Similarly, compounds including pyrocatechol, (−)-epicatechin, taxifolin and 2-hydroxycinnamic acid are reported to be absent in various *Onosma* species such as *O. sieheana*, *O. stenoloba*, *O. isaurica*, *O. gracilis*, *O. aucheriana*, *O. pulchra*, *O. frutescens*, *O. sericea*, *O. ambigens*, and *O. bracteosa* [11,13,20,24,25,26]. Moreover, (+)-catechin and eriodictyol were present in *O. lycaonica* but absent in *O. papillosa*. Similarly, the earlier research on the phytochemical analysis of *Onosma* species has indicated that the compounds such as pyrocatechol, (−)-epicatechin, taxifolin and 2-hydroxycinnamic acid are not observed in *Onosma* species while (+)-catechin and eriodictyol are not commonly observed in *Onosma* species [11,13,20,24,25,26]. Levels of a total of 15 compounds (apigenin, luteolin, eriodictyol, pinoresinol, apigenin 7-glucoside, rosmarinic acid, luteolin 7-glucoside, ferulic acid, vanillin, caffeic acid, 4-hydroxybenzoic acid, (+)-catechin3,4-dihydroxyphenylacetic acid) were found to be high by their concentration in the methanolic extract of *O. lycaonica* compared to *O. papillosa*. These variations in quantity and occurrence of phytochemicals of *Onosma* species is probably due to difference in the extraction methods and ecological conditions of the plant species (climate, soil properties and altitude) [24]. 

### 2.3. Antioxidant Properties 

Most oxidative stress-related human diseases occur due to impairment of the balance between oxidant and antioxidant molecules. The screening of the antioxidant activity of plant extracts provides a basis for discovering novel phytocompounds with bio-health-promoting efficiency. The free radicals such as DPPH, ABTS and phosphomolybdenum are scientifically accepted for the screening of antioxidant molecules from medicinal plants [24,27]. Therefore, the current study screened the free radicals for scavenging activity of two *Onosma* species in comparison with standard antioxidants such as Trolox, ethylenediaminetetraacetic acid, butylated hydroxyanisole, and butylated hydroxytoluene, and the results are shown in Table 3. Phosphomolybdenum scavenging occurs through reduction of Mo (V) to Mo (V) by the interaction of antioxidant molecules from plant extracts or other antioxidant molecules with phosphomolybdenum [28]. In the present work, the methanol extract of the two plants showed a considerable level of phosphomolybdenum scavenging activity, and the EC_50_ value was found to be low for *O. papillosa* (1.90 ± 0.07 mg/mL) as compared to *O. lycaonica* (2.05 ± 0.07 mg/mL). The 1 g extracts of *O. lycaonica* and *O. papillosa* were equivalent to 540.6 ± 19.6 mg and 584.3 ± 20.4 mg of Trolox, respectively. The EC_50_ and TEs values indicted that *O. papillosa* was a stronger phosphomolybdenum scavenging agent as compared with *O. lycaonica*. This is in agreement with earlier reports of phosphomolybdenum scavenging activity of *Onosma* species which showed EC_50_ values in a range of 1.18–2.73 [24,25].

DPPH is a stable free radical and its scavenging reaction occurs through replacement of the nitrogen atom a by hydrogen atom of an oxidant molecules [29]. In the current study, *O. lycaonica* exhibited a stronger DPPH savaging activity than *O. papillosa*, as indicated by a lower IC_50_ value of 2.69 ± 0.10 mg/mL and a higher Trolox equivalent value (92.6 ± 3.6 mg TEs/g extract) of *O. lycaonica* as compared to *O. papillosa* (IC_50_ −3.41 ± 0.05 mg/mL, 73.1 ± 1.0 mg TEs/g extracts). A similar trend of IC_50_ and Trolox equivalent was also observed for ABTS scavenging activity. The IC_50_ and Trolox equivalents of the ABTS+ scavenging significantly varied between the plants studied, with a low IC_50_ (2.18 ± 0.01 mg/mL) and high Trolox equivalent value (130.6 ± 0.4 mg TEs/g extract) for *O. lycaonica*. Thus, *O. lycaonica* was a stronger ABTS+ scavenger as compared to *O. papillosa*. The sample that exhibits less IC_50_, EC_50_ with high Trolox equivalent is considered to be stronger in antioxidant activity than the sample showing high IC_50_, EC_50_ with low Trolox equivalent [11] and this is in accordance with our results on free radical scavenging activity of the *Onosma* species.

Antioxidant molecules scavenge free radicals such as DPPH and ABTS+ through an electron or hydrogen atom transfer mechanism whereas FRAP reduction occurs through an electron reaction related to pH [30]. The methanol extract of *O. lycaonica* showed a low EC_50_ for CUPRAC reduction (1.10 ± 0.01 mg/mL) and FRAP reduction (0.69 ± 0.01 mg/mL) as compared to *O. papillosa*. The EC_50_ of CUPRAC and FRAP reduction along with the Trolox equivalents indicated that *O. papillosa* was potent in reducing the CUPRAC and FRAP. *O. lycaonica* showed higher ferrous ion chelating activity than *O. papillosa*, which was evidenced by the low IC_50_ (2.32 ± 0.16 mg/mL) and high EDTAEs (21.60 ± 1.45 mg EDTAEs/g extract) of *O. lycaonica* (Table 3). Moreover, both plants exhibited low EC_50_ or IC_50_ values compared to standard antioxidants tested such as Trolox, ethylenediaminetetraacetic acid, butylated hydroxyanisole, and butylated hydroxytoluene (Table 3). Overall, the methanol extract of *O. lycaonica* exhibited better antioxidant activity than the *O. papillosa* one, but the phosphomolybdenum scavenging activity difference between the plants was not significant (*p* < 0.05). The higher antioxidant activity of *O. lycaonica* might be attributed to the presence of high levels of antioxidant molecules as evidenced by LC-ESI-MS/MS. The LC-ESI-MS/MS analysis revealed that the total amounts of 15 compounds were found to be higher in *O. lycaonica* than *O. papillosa* (Table 2). Particularly, the antioxidant substances such as apigenin [14], luteolin [31], pinoresinol [32], apigenin 7-glucoside [14], rosmarinic acid [33], ferulic acid [34], vanillin [35], and caffeic acid [36] were found to be higher in the methanol extract of *O. lycaonica*.

### 2.4. Enzyme Inhibition Assay 

The enzyme inhibitory effect of methanol extract of two *Onosma* species (*O. lycaonica* and *O. papillosa*) was assessed for Alzheimer’s disease (AChE, BChE), diabetes (α-amylase and α-glucosidase) and photocarcinogenesis (tyrosinase)-related enzymes by spectrophotometric assays and the results are shown in Table 4. Moreover, the enzyme inhibitory activity was compared to standard enzyme inhibitors such as galantamine for AChE, and BChE, acarbose for α-amylase and α-glucosidase, kojic acid for tyrosinase. 

The AChE and BChE inhibition activities varied significantly between the plants, but did not vary for α-glucosidase, α-amylase, and tyrosinase inhibition activities (*p* < 0.05). *O. lycaonica* exhibited high AChE inhibition activity (IC_50_ −1.32 ± 0.02 mg/mL) while *O. papillosa* showed high BChE inhibition (IC_50_ −4.96 ± 0.07 mg/mL). Thus, both plants showed potent inhibition of the targeted enzymes. Although the IC_50_ and standard galantamine equivalent, acarbose equivalent and kojic acid equivalent did not vary between the two *Onosma* species analyzed in the present study, an earlier work has reported significant variations between the enzyme inhibitory activity of *O. sericea* and *O. stenoloba* [12]. Thus the methanol extract of *Onosma* species is a promising source for the isolation of enzyme inhibitors, as disclosed in earlier reports [12,22,24].

## 3. Materials and Methods 

### 3.1. Standard Phytochemicals and Chemicals

2,5-Dihydroxybenzoic acid, pyrocatechol, chlorogenic acid, 4-hydroxybenzoic acid, (−)-epicatechin, caffeic acid, gallic acid, (+)-catechin, vanillin, syringic acid, taxifolin, *p*-coumaric acid, sinapic acid, ferulic acid, 2-hydroxycinnamic acid, rosmarinic acid, pinoresinol, luteolin, quercetin, apigenin and HPLC grade of methanol and formic acid were obtained from Sigma-Aldrich (St. Louis, MO, USA). 3-Hydroxybenzoic acid, vanillic acid, apigenin 7-glucoside, 3,4-dihydroxyphenylacetic acid, luteolin 7-glucoside, eriodictyol, hesperidin, and kaempferol were obtained from Fluka (St. Louis, MO, USA). The hyperoside protocatechuic acid and verbascoside were purchased from HWI Analytik (Ruelzheim, Germany). Ultra-pure water (18.2 mΩ/cm) was prepared by using a Milli-Q water purification system (Milli-Q Millipore Merck KGaA Darmstadt, Germany). All the chemicals and reagents used in the biological assay were obtained from Sigma-Aldrich. 

### 3.2. Plant Material and Extract Preparation

*O. lycaonica* Hub. -Mor. and *O. papillosa* Riedl were collected from Sertavul Pass, Mut, Mersin-Turkey (1660 m., 36°54′18″ N 33°16′14″ E, herbarium number: OC.5057) and the Yesilkent-Tufanbeyli highway, Tufanbeyli-Adana (1540 m., 38°15′58″ N 36°20′54″ E, herbarium number: OC.5058), respectively. These plant species were identified and authenticated by Dr. Olcay Ceylan (Mugla Sitki Kocman University). The aerial parts of the samples were separated, and shadow air-dried for several weeks without direct exposure of sunlight. Afterwards, these samples were cut into small pieces. The plant sample weighed at 5 g was immersed in 100 mL of methanol at ambient room condition for 24 h and filtered using the Whatman No.1. filter paper. The extraction was repeated two times and the extracts were pooled together for each plant species, and concentrated using a rotary evaporator [11] and the extracts were preserved at 4 °C for further experiments.

### 3.3. Analysis of Total Phenolics and Flavonoids 

The content of total phenolics in the plant extracts was determined by spectrophotometric assay using Folin-Ciocalteu reagent according to the methods described earlier [37,38]. In brief, 0.25 mL of plant extract was mixed with Folin-Ciocalteu reagent (1 mL, 1:9) and vigorously vortexed for 3 min followed by 0.75 mL of 1% Na_2_CO_3_ were added and incubated for 2 h in room temperature. After incubation, the sample was measured for optical density (OD) at 760 nm, and the content of total phenolics is presented as gallic acid equivalents. The total flavonoids in the extracts were measured according to the methods described earlier [39]. In brief, the 2% of aluminum chloride solution was prepared in methanol. Then the plant extract was mixed with AlCl_3_ solution at 1:1 ratio. A blank, prepared by mixing methanol with AlCl_3_ at the same ratio. The plant and blanks were incubated at room temperature for 10 min then the OD was measured at 415 nm, and the content of flavonoids is presented as quercetin equivalents.

### 3.4. Analysis of Phytochemical Composition by LC–ESI–MS/MS

The selected phytochemical constituents were analysed in the methanolic extracts of *Onosma* spp., by using LC–ESI–MS/MS (1260 Infinity liquid chromatography system hyphenated to a 6420 Triple Quad mass spectrometer, Agilent Technologies, Santa Clara, CA, USA) equipped with Poroshell 120 EC-C18 (100 mm × 4.6 mm I.D., 2.7 μm) column. In order to analyze the compounds, the mobile phases were prepared using different combinations of formic acid, ammonium acetate, methanol and acetic acid according to target compounds isomeric resolution as described in our earlier study [40]. The LC-ESI-MS/MS analysis was operated according to the methods described earlier [40].

### 3.5. Antioxidant, and Enzyme Inhibition Assays

The antioxidant activities of methanolic extracts of two *Onosma* spp., were examined according to the protocols described previously [41,42,43,44,45] by using three scavenging assays by using phosphomolybdenum, 1,1-diphenyl-2-picrylhydrazyl (DPPH) and 2,2-azino-bis (3-ethylbenzothiazloine-6-sulphonic acid, ABTS+), and two reducing assays measuring the cupric ion reducing (CUPRAC) and ferric reducing (FRAP) properties, and one chelating assay using ferrous ion chelation. The plant extracts were also examined for inhibition of enzymes such as α-glucosidase, α-amylase, acetylcholinesterase (AChE), butyrylcholinesterase (BChE) and tyrosinase, which are related to diabetes, Alzheimer’s disease and photocarcinogenesis, according to the methods described elsewhere [23]. The results are presented as IC_50_ values and the EC_50_ values are calculated using a formula described earlier [41].

### 3.6. Statistical Analysis

Biological assays and phytochemical composition analysis were performed for three times. The statistical analysis such as descriptive statistics, and one-way ANOVA and post hoc test (Tukey’s) and student *t*-test were performed to observe the significance (*p* < 0.05) between extracts (*O. lycaonica* and *O. papillosa*) by using statistical software package SPSS v. 22.0 (PSS Inc, Chicago, IL, USA). 

## 4. Conclusions

This work reports the phytochemical composition, antioxidant and enzyme inhibitory activity of two *Onosma* species endemic to Turkey. The methanol extract of *O. lycaonica* exhibited high levels of ~15 antioxidant-related compounds and antioxidant activities as compared to *O. papillosa*. Both plant species showed considerable enzyme inhibitory activity. The present results evidenced that these endemic *Onosma* species (*O. lycaonica* and *O. papillosa*) represent promising sources for the isolation of pharmacologically important drug candidate molecules to treat various oxidative diseases, including diabetes, cancer, Alzheimer’s disease and photocarcinogenesis.

## Figures and Tables

**Table 1:** Extraction yield, total flavonoid and phenolic contents of *O. lycaonica* and *O. papillosa* extracts. QEs and GAEs: Quercetin and gallic acid equivalents, respectively. The differ superscript in row indicates the significance between the species post hoc (Tukey’s test; *p* < 0.05).

Assays	*O. lycaonica*	*O. papillosa*
Yield (%)	3.52	4.02
Total flavonoids (mg QEs/g extracts)	26.0 ± 0.5 ^b^	32.9 ± 0.3 ^a^
Total phenolics (mg GAEs/g extracts)	43.5 ± 1.5 ^a^	33.9 ± 0.4 ^b^

**Table 2:** Concentration (µg/g extract) of selected phenolic compounds in *O. lycaonica* and *O. papillosa* extracts. nd: Not detected. The differ superscript in row indicates the significance between the species post hoc (Tukey’s test; *p* < 0.05).

Compound	*O. lycaonica*	*O. papillosa*
Gallic acid	12.6 ± 0.4 ^b^	15.2 ± 0.1 ^a^
Protocatechuic acid	162.2 ± 3.6 ^b^	249 ± 1.9 ^a^
3,4-Dihydroxyphenylacetic acid	11.1 ± 0.6 ^a^	7.4 ± 0.1 ^b^
(+)-Catechin	30.4 ± 4.2	nd
Pyrocatechol	nd	nd
Chlorogenic acid	847.5 ± 19.5 ^b^	14088 ± 115 ^a^
2,5-Dihydroxybenzoic acid	209.5 ± 4.5 ^b^	265.8 ± 11.0 ^a^
4-Hydroxybenzoic acid	953.4 ± 7.6 ^a^	935.3 ± 2.7 ^a^
(−)-Epicatechin	nd	nd
Caffeic acid	863.0 ± 43.2 ^a^	222.2 ± 2.8 ^b^
Vanillic acid	1260 ± 25 ^a^	622.1 ± 12.1 ^b^
Syringic acid	30.1 ± 1.6 ^b^	74.3 ± 0.4 ^a^
3-Hydroxybenzoic acid	21.0 ± 1.2 ^a^	17.6 ± 0.1 ^a^
Vanillin	80.7 ± 3.9 ^a^	70.2 ± 6.8 ^a^
Verbascoside	nd	nd
Taxifolin	nd	nd
Sinapic acid	26.8 ± 2.8 ^b^	83.1 ± 2.1 ^a^
*p*-Coumaric acid	189.5 ± 1.6 ^b^	226.7 ± 0.3 ^a^
Ferulic acid	1204 ± 8 ^a^	495.6 ± 21.6 ^b^
Luteolin 7-glucoside	20,846 ± 522 ^a^	789.1 ± 10.4 ^b^
Hesperidin	15,417 ± 288 ^b^	54,123 ± 239 ^a^
Hyperoside	2853 ± 64 ^b^	4555 ± 100 ^a^
Rosmarinic acid	65,632± 1418 ^a^	6312 ± 110 ^b^
Apigenin 7-glucoside	21,416 ± 361 ^a^	1217 ± 129 ^b^
2-Hydroxycinnamic acid	nd	nd
Pinoresinol	3567 ± 8 ^a^	2756 ± 49 ^b^
Eriodictyol	4.3 ± 0.2	nd
Quercetin	11.0 ± 0.2 ^b^	49.6 ± 0.3 ^a^
Luteolin	2559 ± 46 ^a^	277.4 ± 20.4 ^b^
Kaempferol	nd	42.2 ± 2.6
Apigenin	1623 ± 34 ^a^	319.6 ± 18.9 ^b^

**Table 3:** Antioxidant activity of *O. lycaonica* and *O. papillosa* extracts. TEs and EDTA Es mean trolox and ethylenediaminetetraacetic acid (disodium salt) equivalents, BHA (butylated hydroxyanisole), BHT (butylated hydroxytoluene). The differ superscript in row indicates the significance between the species post hoc (Tukey’s test; *p* < 0.05).

Antioxidant Activity	*O. lycaonica*	*O. papillosa*	Trolox	BHA	BHT	EDTA
Phosphomolybdenum (EC_50_: mg/mL)	2.05 ± 0.07 ^c^	1.90 ± 0.07 ^c^	1.16 ± 0.06 ^b^	0.31 ± 0.01 ^a^	0.38 ± 0.03 ^a^	
DPPH scavenging (IC_50_: mg/mL)	2.69 ± 0.10 ^c^	3.41 ± 0.05 ^d^	0.26 ± 0.02 ^a^	0.22 ± 0.02 ^a^	1.00 ± 0.03 ^b^	
ABTS scavenging (IC_50_: mg/mL)	2.18 ± 0.01 ^b^	2.50 ± 0.16 ^c^	0.32 ± 0.03 ^a^	0.21 ± 0.01 ^a^	0.29 ± 0.02 ^a^	
CUPRAC reducing (EC_50_: mg/mL)	1.10 ± 0.01 ^c^	1.32 ± 0.02 ^d^	0.28 ± 0.02 ^b^	0.14 ± 0.01 ^a^	0.18 ± 0.02 ^a^	
FRAP reducing (EC_50_: mg/mL)	0.69 ± 0.01 ^c^	0.88 ± 0.02 ^d^	0.10 ± 0.01 ^a^	0.09 ± 0.01 ^a^	0.18 ± 0.01 ^b^	
Ferrous ion chelating (IC_50_: mg/mL)	2.32 ± 0.16 ^b^	3.65 ± 0.07 ^c^				0.051 ± 0.003 ^a^
						
Phosphomolybdenum (mg TEs/g extracts)	540.6 ± 19.6 ^a^	584.3 ± 20.4 ^a^				
DPPH scavenging (mg TEs/g extracts)	92.6 ± 3.6 ^a^	73.1 ± 1.0 ^b^				
ABTS scavenging (mg TEs/g extracts)	130.6 ± 0.4 ^a^	115.1 ± 7.1 ^a^				
CUPRAC reducing (mg TEs/g extracts)	249.5 ± 1.4 ^a^	207.5 ± 3.9 ^b^				
FRAP reducing (mg TEs/g extracts)	144.4 ± 0.1 ^a^	113.1 ± 0.1 ^b^				
Ferrous ion chelating (mg EDTAEs/g extracts)	21.6 ± 1.5 ^a^	14.0 ± 0.3 ^b^				

**Table 4:** Enzyme inhibition activity of *O. lycaonica* and *O. papillosa* extracts. GALAEs, KAEs and ACEs mean galanthamine, kojic acid and acarbose equivalents, respectively. The differ superscript in row indicates the significance between the species post hoc (Tukey’s test; *p* < 0.05).

Enzyme Inhibitory Activity	*O. lycaonica*	*O. papillosa*	Galanthamine	Acarbose	Kojic Acid
AChE inhibition (IC_50_: mg/mL)	1.32 ± 0.02 ^b^	1.47 ± 0.06 ^c^	0.0035 ± 0.0004 ^a^	-	-
BChE inhibition (IC_50_: mg/mL)	8.94 ± 0.08 ^c^	4.96 ± 0.07 ^b^	0.0058 ± 0.0003 ^a^	-	-
α-Amylase inhibition (IC_50_: mg/mL)	2.57 ± 0.11 ^b^	2.40 ± 0.07 ^b^	-	0.97 ± 0.03 ^a^	-
α-Glucosidase inhibition (IC_50_: mg/mL)	2.60 ± 0.14 ^b^	2.61 ± 0.04 ^b^	-	1.74 ± 0.03 ^a^	-
Tyrosinase inhibition (IC_50_: mg/mL)	2.20 ± 0.03 ^b^	2.05 ± 0.08 ^b^	-	-	0.31 ± 0.01 ^a^
					
AChE inhibition (mg GALAEs/g extracts)	2.31 ± 0.04 ^a^	2.07 ± 0.08 ^a^			
BChE inhibition (mg GALAEs/g extracts)	0.63 ± 0.01 ^b^	1.13 ± 0.02 ^a^			
α-Amylase inhibition (mgACEs/g extracts)	402.9 ± 18.1 ^a^	430.7 ± 13.5 ^a^			
α-Glucosidase inhibition (mgACEs/g extracts)	670.4 ± 36.7 ^a^	666.7 ± 10.7 ^a^			
Tyrosinase inhibition (mg KAEs/g extracts)	139.0 ± 1.9 ^a^	149.0 ± 5.6 ^a^			

## Data Availability

The data presented in this study are available in the article.

## Supplementary Materials

The following are available online at https://www.mdpi.com/article/10.3390/plants10071373/s1, Table S1: ESI–MS/MS Parameters and analytical characteristics for the Analysis of target analytes by MRM Negative and Positive Ionization Mode, Table S2: Calibration curves and sensitivity properties of the method.
